# Expert consensus on endodontic therapy for patients with systemic conditions

**DOI:** 10.1038/s41368-024-00312-0

**Published:** 2024-06-17

**Authors:** Xin Xu, Xin Zheng, Fei Lin, Qing Yu, Benxiang Hou, Zhi Chen, Xi Wei, Lihong Qiu, Chen Wenxia, Jiyao Li, Lili Chen, Zuomin Wang, Hongkun Wu, Zhiyue Lu, Jizhi Zhao, Yuhong Liang, Jin Zhao, Yihuai Pan, Shuang Pan, Xiaoyan Wang, Deqin Yang, Yanfang Ren, Lin Yue, Xuedong Zhou

**Affiliations:** 1 State Key Laboratory of Oral Diseases & National Clinical Research Center for Oral Diseases & Department of Cariology and Endodontics, West China Hospital of Stomatology, Sichuan University, Chengdu, China; 2 Department of Cariology and Endodontology, Peking University School and Hospital of Stomatology & National Clinical Research Center for Oral Diseases & National Engineering Research of Oral Biomaterials and Digital Medical Devices & Beijing Key Laboratory of Digital Stomatology, Peking University, Beijing, China; 3 State Key Laboratory of Oral & Maxillofacial Reconstruction and Regeneration, National Clinical Research Center for Oral Diseases, Shaanxi Key Laboratory of Oral Diseases, Department of Operative Dentistry & Endodontics, School of Stomatology, The Fourth Military Medical University, Xi’an, China; 4 Center for Microscope Enhanced Dentistry, Beijing Stomatological Hospital, Capital Medical University, Beijing, China; 5 State Key Laboratory of Oral & Maxillofacial Reconstruction and Regeneration, Key Laboratory of Oral Biomedicine Ministry of Education, Hubei Key Laboratory of Stomatology, School & Hospital of Stomatology, Wuhan University, Wuhan, China; 6 Department of Operative Dentistry and Endodontics, Hospital of Stomatology, Guanghua School of Stomatology, Sun Yat-Sen University & Guangdong Provincial Key Laboratory of Stomatology, Guangzhou, China; 7 Department of Endodontics, School of Stomatology, China Medical University, Shenyang, China; 8 College & Hospital of Stomatology, Guangxi Medical University, Nanning, China; 9 Department of Stomatology, Union Hospital, Tongji Medical College, Huazhong University of Science and Technology, Wuhan, China; 10 Department of Stomatology, Beijing Chaoyang Hospital, Capital Medical University, Beijing, China; 11 State Key Laboratory of Oral Diseases & National Center for Stomatology & National Clinical Research Center for Oral Diseases & Department of Geriatric Dentistry, West China Hospital of Stomatology, Sichuan University, Chengdu, China; 12 Beijing Hospital, National Center of Gerontology, Beijing, China; 13 Department of Stomatology, Peking Union Medical College Hospital, Chinese Academy of Medical Sciences & Peking Union Medical College, Beijing, China; 14 Department of Emergency, Peking University School and Hospital of Stomatology & National Center of Stomatology & National Clinical Research Center for Oral Diseases & National Engineering Laboratory for Digital and Material Technology of Stomatology & Beijing Key Laboratory of Digital Stomatology & Research Center of Engineering and Technology for Computerized Dentistry Ministry of Health & NMPA Key Laboratory for Dental Materials, Beijing, China; 15 Department of Endodontics, The First Affiliated Hospital of Xinjiang Medical University, Urumqi, China; 16 Department of Endodontics, School and Hospital of Stomatology, Wenzhou Medical University, Wenzhou, China; 17 Department of Endodontics, The First Affiliated Hospital of Harbin Medical University & Department of Endodontics, School of Stomatology, Harbin Medical University, Harbin, China; 18 Department of Conservative Dentistry and Endodontics, Shanghai Stomatological Hospital & School of Stomatology, Fudan University, Shanghai, China; 19 Shanghai Key Laboratory of Craniomaxillofacial Development and Diseases, Fudan University, Shanghai, China; 20 Eastman Institute for Oral Health, University of Rochester Medical Center, Rochester, NY USA

**Keywords:** Pulpitis, Endodontics

## Abstract

The overall health condition of patients significantly affects the diagnosis, treatment, and prognosis of endodontic diseases. A systemic consideration of the patient’s overall health along with oral conditions holds the utmost importance in determining the necessity and feasibility of endodontic therapy, as well as selecting appropriate therapeutic approaches. This expert consensus is a collaborative effort by specialists from endodontics and clinical physicians across the nation based on the current clinical evidence, aiming to provide general guidance on clinical procedures, improve patient safety and enhance clinical outcomes of endodontic therapy in patients with compromised overall health.

## Introduction

Classic endodontic therapy can be categorized into nonsurgical endodontic therapy (NSET) and surgical endodontic therapy (SET). NSET primarily involves root canal treatment and retreatment, while SET mainly focuses on performing apical surgery to address persistent periapical lesions when nonsurgical retreatment fails or cannot be performed. When indicated, techniques like guided tissue regeneration may also be employed to facilitate the regenerative repair of periapical lesions and preserve the function of affected teeth. Prior to commencing endodontic therapy, it is imperative to conduct a comprehensive exam to evaluate the patient’s systemic and oral health conditions in addition to the affected tooth. This step is important in determining the safety, necessity, and feasibility of treatment, as well as selecting appropriate therapeutic approaches. Extensive research has consistently demonstrated that various systemic factors such as age, pregnancy, cardiovascular diseases, bleeding disorders, chronic metabolic conditions like diabetes, malignant tumors, communicable infectious diseases such as hepatitis and AIDS can all affect the treatment plan for the affected tooth (e.g., conservative management vs. extraction, NSET vs. SET, anesthesia technique and drug selection, prophylactic antibiotic use, and temporary discontinuation of long-term medications). These systemic conditions may also have impacts on diagnosis, treatment complexity, and prognosis of endodontic diseases. Endodontists must possess fundamental knowledge on the interplays between systemic factors and endodontic diseases and understand the key clinical management aspects in treating patients with systemic conditions. This expert consensus was developed by experts in endodontics across the nation after multiple rounds of deliberations through online and in person conferences regarding the consideration of systemic factors in endodontic therapy. By fostering consensus, this initiative aimed to offer general guidance for the diagnosis and treatment of endodontic diseases in patients with systemic conditions. It is important to note that this consensus is not intended to establish a standard of care, nor is it intended to deprive dentists of their abilities to exercise their own professional judgment in clinical decision-making. The primary goal was to enhance the functional preservation of teeth to the fullest extent while ensuring no compromise to overall health. Due to the typically urgent nature of endodontic therapy in pregnant woman and the potential need for interprofessional care,^1^ a dedicated consensus will address this topic, and consequently it is not covered in the present work.

This consensus is based on currently available evidence and primarily provides general guidance for non-emergency endodontic therapy. In cases of endodontic emergencies, the dental practitioners should keep in mind that dental procedures are aimed to alleviate acute pain timely and reduce the risk of disease progressions that may further compromise the systemic conditions. Emergency dental procedures such as pulpectomy for symptomatic irreversible pulpitis or incision and drainage for pulp necrosis with localized acute apical abscess should be performed as soon as possible after thorough assessment of the patient’s overall health conditions. If such emergency procedures are deemed unsafe for patients with severe systemic conditions, the patient should be prescribed analgesics to alleviate pain and be referred immediately to physicians and/or hospital dental clinics that could provide urgent dental treatments in a timely fashion.

## Clinical considerations for endodontic therapy in patients with systemic conditions

### Cardiovascular disease

Due to aging of the population and changes in lifestyle, the incidence of cardiovascular disease (CVD) has been steadily increasing over time, positioning it as one of the leading global causes of mortality.^2–4^ Therefore, during diagnosis and treatment procedures, meticulous attention should be paid to whether the patient has CVD in order to prevent potential adverse consequences throughout the therapeutic process.

During the diagnostic process, it is crucial to distinguish between odontogenic pain and cardiac pain referred to tooth/teeth. This type of toothache represents an early indication of occult coronary heart disease.^5,6^ Due to its potentially life-threatening consequences, it is crucial to give the highest level of attention to cardiac pain that is referred to the tooth or teeth. Typical characteristics of cardiac referred pain include: (1) sudden and severe toothache, mostly affecting the mandibular teeth, but without any evident pulpitis, apical periodontitis, or other dental issues that can cause toothache^7^; (2) craniofacial pain as a “tightness” and “burning sensation,” different from the “throbbing” and “stabbing” pain of dental origin^6^; (3) toothache accompanied by chest tightness, chest pain, or shoulder/back pain^7^; (4) toothache triggered by exertion or emotional excitement, which can be relieved by rest or taking nitroglycerin but not by painkillers or tooth extraction^7,8^; (5) patients with cardiovascular risk factors such as obesity, hypertension, hyperlipidemia, diabetes and etc., especially elderly individuals experiencing unexplained toothache. Patients diagnosed with or suspected of cardiac pain referred to tooth/teeth should be advised to promptly seek medical attention from a cardiologist.

Patients with poorly controlled severe CVD should refrain from undergoing elective endodontic therapy. Specific situations that warrant caution include recent myocardial infarction within 6 months, congestive heart failure, unstable angina pectoris, uncontrolled arrhythmia, inadequately managed hypertension, and cardiac-related surgery performed within 6 months.^9,10^ For patients with relatively stable CVD, treatment should be administered following a thorough assessment of the patient’s medical history. On the day of treatment, the patient should have no symptoms such as chest pain, headache, dizziness, and are emotionally calm and relaxed without anxiety. Patients with a history of hypertension should undergo routine blood pressure measurement prior to treatment. Ideally, the patient’s blood pressure should be controlled below 140 mmHg/90 mmHg. In non-emergency situation, if the systolic pressure reaches 180 mmHg or the diastolic pressure reaches 110 mmHg, it is advisable for patients to stabilize their blood pressure levels before undergoing endodontic therapy. For values in between, evaluation can be based on overall health condition and treatment urgency.^11,12^ There is no need to stop anticoagulant medications before NSET.^9,13,14^ However, as patients are advised to stop taking warfarin 2–3 days before surgical dental procedures,^15,16^ it is also applicable to patients undergoing SET to avoid serious bleeding complications during and after the procedure.

Pain or anxiety can trigger the release of endogenous catecholamines in individuals with CVD, potentially increasing the risk of adverse cardiovascular events.^17^ Hence, it is crucial to focus on pain management, anxiety alleviation, and judicious use of local anesthetics during endodontic therapy for patients with CVD. Adding vasoconstrictors such as epinephrine to local anesthetics can effectively slow drug absorption, reduce toxic reactions, extend the duration of local anesthesia, and minimize bleeding in the surgical area to ensure a clear field of vision.^18^ A recent systematic review claimed that use of epinephrine in low doses (1:100 000) does not alter cardiovascular risks such as arrhythmia and elevated blood pressure, suggesting safety in its use for patients with CVD in dental procedures.^19^ Nevertheless, when directly injected into blood vessels during nerve blocks or administered in relatively high doses (exceeding 0.04 mg) in a short time span during submucosal infiltration,^20^ epinephrine may induce systemic effects on blood pressure and heart rate, which should be avoided. Local anesthetics contain minimal amounts of epinephrine (not exceeding 0.0225 mg) are well tolerated by patients with CVD classified as functional grades I–III.^21^

Patients with artificial heart valves, past infective endocarditis (IE) and heart transplant are at risk of developing IE during invasive endodontic procedures that involve manipulation of the gingival tissue or the periapical region, or perforation of the oral mucosa.^22^ The prophylactic administration of antibiotics prior to invasive endodontic therapies involving the apical tissues can effectively minimize the potential for serious cardiac complications.^22^ For high-risk patients, it is recommended to take oral amoxicillin (2 000 mg) 1 h before the procedure. In case of allergy, a macrolide should be used as an alternative.^22^

Currently, there is no consensus regarding the relationship between CVD and the outcome of endodontic therapy.^23,24^ Several studies have reported a significant correlation between systemic effects of diabetes and CVD and failure of endodontic therapy.^25,26^ Researchers have proposed that both apical periodontitis and CVD are associated with an elevated systemic inflammatory burden. Consequently, systemic diseases linked to inflammation may impair crucial immune factors involved in the pathogenesis of apical periodontitis,^27^ potentially impacting the outcome of the treatment. Most acquired CVD are associated with increased systemic inflammatory burdens, which can lead to adverse outcomes in endodontic therapy,^28,29^ and may be a risk factor for treatment failure.^30^ However, Laukkanen et al.^31^ found that CVD did not have a significant effect on the success rate of endodontic therapy. It is worth noting that this study did not conduct stratified analysis based on specific types of CVD such as hypertension, coronary artery disease, and valvular heart disease.^31^

For patients with CVD, the expert consensus panel recommend the following:

- Reschedule non-emergency treatment for patients with unstable CVD (within 6 months after myocardial infarction, congestive heart failure, unstable angina pectoris, uncontrolled arrhythmias, poorly controlled hypertension, or within 6 months after cardiac-related surgery) until CVD is properly managed.
- Consider prophylactic use of antibiotics on high-risk patients (patients with artificial heart valves, past infective endocarditis, and heart transplant) prior to invasive endodontic therapies to minimize the risk of serious cardiac complications.
- Pay attention to pain management, anxiety alleviation, and judicious use of local anesthetics during endodontic therapy for patients with CVD.
- Perform emergency dental procedures, such as pulpectomy for symptomatic irreversible pulpitis or incision and drainage for localized acute apical abscess as soon as possible, after a thorough assessment of the patient’s overall health conditions.
- Make efforts to distinguish cardiac pain referred to tooth/teeth from toothache from endodontic origins.

### Diabetes mellitus

Diabetes mellitus can impact the occurrence, development, and prognosis of endodontic diseases.^32^ The endodontists should be familiar with the medical management of diabetes. Referral to an endocrinologist should be considered for patients with inadequate blood glucose control or those experiencing severe complications such as renal disease, hypertension, or coronary artery atherosclerosis.^33^ For insulin-dependent patients, severe hypoglycemia may occur if the patient uses insulin but is not able to eat a meal due to severe dental pain. If a patient’s blood glucose level is below 37 mg/dL, they should consume ~15 g of carbohydrates before undergoing endodontic therapy, such as a glass of juice or a few cookies. Scheduling morning appointments can help avoid insulin peaks, and patients should follow the physician’s instructions regarding medication intake and breakfast when visiting dentists for endodontic therapy.^34,35^

For diabetic patients who require NSET, prophylactic use of antibiotics is generally unnecessary. For SET, recommendations are mainly based on available evidence for tooth extraction in diabetic patients. In cases where diabetic patients with poor oral hygiene require invasive treatment, prophylactical administration of antibiotics could be considered when fasting blood glucose levels exceed 200 mg/dL in order to prevent bacteremia.^36^ Treatment duration exceeding 3–4 h may require antibiotic prophylaxis prior to surgery and administration of antibiotics during the procedure to reduce the risk of developing severe postoperative infections.^36^

For local anesthesia, routine use of local anesthetic with 1:100 000 epinephrine is generally well tolerated for type II diabetic patient.^37^ Particular care should be taken in patients with type I diabetes treated with high doses of insulin, especially those with unstable blood glucose levels, and the use of vasoconstrictors should be minimized due to the potential for vasoconstrictor-enhanced hypoglycemia.^38^

Diabetes can result in delayed healing of periapical lesions and is often accompanied by periodontal diseases, which may lead to poor prognosis for affected teeth.^39–41^ Periodontal diseases also impact the outcome of endodontic therapy.^42^ Regular follow-ups are recommended for patients who have undergone endodontic therapy to allow early identification and treatment of periodontal diseases.

For patients with diabetes, the expert consensus panel recommend the following:

- Pay close attention to glycemic control and periodontal health before and after endodontic therapy.
- Schedule endodontic therapy in the morning for patients with advanced diabetes.
- Be cautious of the risk of hypoglycemia in insulin-dependent patients.
- Consider prophylactic antibiotics for patients with poor glycemic control (blood glucose > 200 mg/dL) and severe endodontic or systemic infections.

### Chronic kidney disease

Many patients with chronic kidney disease (CKD) are asymptomatic in the early stages, and typical clinical features only manifest in the advanced stage of CKD. Clinical symptoms of renal failure involve multiple body systems, including uremia, renal malnutrition, asterixis, coagulation disorders, congestive heart failure, and hypertension.^43^ Severe CKD may affect organs and tissues in the oral cavity, including salivary glands, periodontal tissues, teeth, alveolar bone, and mucosa, leading to issues such as gingival bleeding, early tooth loss, periodontitis, and xerostomia,^44^ which may in turn affect the outcome of endodontic therapy.

Prior to invasive SET, it is advisable to evaluate the patient’s blood clotting function and blood pressure in patients with advanced CKD. Due to impaired kidney function and platelet dysfunction in advanced CKD patients, there might be an increased risk of bleeding tendency. Preoperative assessment of clotting function is therefore helpful in treatment planning for these patients.^44^ As renal insufficiency often coexists with hypertension, preoperative blood pressure measurement should also be conducted.^45^

Kidney transplant recipients are at a higher risk of organ rejection and often receive high doses of immunosuppressants, rendering them highly susceptible to infections.^46^ It is recommended that post-kidney transplant patients refrain from elective endodontic therapy within the first 6 months.^47^

During treatment, it is recommended to consult a nephrologist regarding the use of antibiotics to manage oral infectious diseases in patients with end stage CKD. Typically, penicillin or cephalosporins are recommended for patients without history of drug allergies, while tetracycline and streptomycin should be avoided due to their potential nephrotoxicity. Intravenous administration of antibiotics is preferred in cases of renal impairment and gastrointestinal malabsorption. For local anesthesia, it is recommended to use an amide anesthetic such as lidocaine due to its hepatic reabsorption potential.^48^ Non-steroidal anti-inflammatory drugs (NSAIDs) have long been regarded as risky for CKD patients due to their potential for nephrotoxicity.^49^

For patients with CKD, the expert consensus panel recommend the following:

- Pay attention to coagulation function and blood pressure in patients with end stage CKD.
- Avoid using nephrotoxic drugs, including tetracycline, streptomycin, and NSAIDs.
- Carefully consider the timing of endodontic therapy in non-emergency situations. Avoid elective endodontic therapy within the first 6 months after renal transplantation.

### Blood-borne transmissible infectious diseases

Meticulous attention should be given to infection control during treatment for all endodontic patients considering the increased prevalence of blood-borne transmissible infectious diseases such as hepatitis and HIV/AIDS. Many patients may not be aware or may not disclose that they have transmissible infectious diseases. Therefore, it is important for dental practitioners to adopt a standard precaution strategy, that is, to treat every patient in the dental chair as having a blood-borne transmissible infectious disease.^50–52^ Because, hepatitis A and other diseases can be transmitted through saliva, hepatitis B or C, AIDS/HIV, and other diseases can be transmitted through blood, endodontic therapy is a potential route of transmission. For patient diagnosed with advanced stage of transmissible infectious diseases such as AIDS or hepatitis B or C, it is advisable for endodontists to be aware of the patient’s coagulation function, immune suppression, medication status, and susceptibility to opportunistic infections. After completing the treatment, thorough surface disinfection of treatment area should be carried out while instruments are strictly cleaned and sterilized. Appropriate personal protective equipment such as gloves, eye protection goggles, face shields, and isolation gowns should be worn. Additionally, it is important to avoid mixed-mode transmission through contact with wounds in order to prevent inadvertent cross-infection.^53^

Generally, AIDS/HIV patients with a CD4^+^ cell count higher than 350/mm^3^ can safely undergo all dental treatments. However, patients with a CD4^+^ cell count lower than 200/mm^3^ or severe neutropenia (neutrophil count below 500/μL) have an increased risk of opportunistic infections. In such cases, endodontists may consider using prophylactic antibiotics or antimicrobials based on the treatment plan.^54,55^ Postoperative medication use should also consider potential adverse reactions including hepatotoxicity, nephrotoxicity, immunosuppression, and drug interactions. When examining the patients and formulating treatment plans, endodontists should also be aware of other oral manifestations of AIDS/HIV infection such as tuberculous ulcer, oral candidiasis, hairy leukoplakia, Kaposi’s sarcoma, linear gingival erythema, and necrotizing ulcerative gingivitis. Further modifications to the treatment plan should be made based on the patient’s symptoms and overall conditions. To date, no study has suggested that AIDS/HIV impacts the outcome of endodontic therapy. With the widespread use of highly active antiretroviral therapy (HAART) and combination antiretroviral therapy, AIDS patients often have CD4^+^ cell and neutrophil counts close to normal levels.^56^ Several retrospective and prospective studies have consistently shown that endodontic therapy on HIV-positive patients has comparable clinical outcome to HIV-negative individuals.^57–59^

For patients with known infectious liver diseases undergoing SET, it is advisable to conduct a comprehensive blood panel assessment and assess coagulation function to understand potential risks for intraoperative bleeding. Hepatotoxic drugs, such as acetaminophen, tetracyclines, sulfonamide antibiotics, cefoperazone/sulbactam, and certain fluoroquinolones should be avoided, or their dosages be adjusted. Penicillin, cefotaxime sodium (cefotaxime), ceftazidime (cephalosporin), vancomycin are primarily eliminated through renal excretion and generally do not necessitate dose adjustment.^60^ Patients with advanced liver disease often exhibit systemic symptoms such as malnutrition, infection, and ascites. Therefore, to improve the outcome of endodontic therapy, preventive measures such as the use of hemostatic drugs can be considered to reduce complications associated with invasive endodontic therapy.^61,62^

It is imperative for dental practitioners to be aware that blood-borne transmissible infectious diseases are common amongst the dental patients, and that the patients may not know or may not disclose that they have these diseases. Hence, the expert consensus panel recommend the following:

- Adopt a standard precaution strategy of infection control and use proper personal protective equipment during dental procedures.
- Pay attention to coagulation function and immune suppression in patients with infectious liver diseases or active HIV/AIDS.
- Consider using prophylactic antibiotics in HIV/AIDS patients with CD4^+^ < 200/mm^3^ or neutrophil below 500/μl.
- Avoid using hepatotoxic drugs in patients with infectious liver diseases.

### Autoimmune diseases

Patients with autoimmune diseases, such as Henoch–Schönlein purpura (HSP), rheumatic diseases and autoimmune hepatitis/nephritis, may have a higher risk of developing endodontic diseases.^63–68^ It is important to make efforts to prevent odontogenic infections during the treatment to avoid exacerbating abnormal immune responses. For example, proactive measures should be taken to minimize risks for caries or endodontic diseases in HSP children to prevent the oral cavity from becoming a primary source of infection for other organs in the body. These measures include improving oral hygiene and dietary habits and applying fluoride treatments and pit and fissure sealant. For children who have already developed endodontic diseases, especially for those with severe renal dysfunction, it is crucial to eliminate active infectious lesions in the oral cavity to prevent dental-related infections and facilitate the prevention and treatment of HSP.^69^ Prior to endodontic therapy, prophylactic use of antibiotics should be considered to minimize the risk of inducing HSP through bacteremia induced by dental procedures.^70,71^

There is no consensus on the impact of immunosuppressive drugs on the treatment outcome of endodontic therapy in current literature,^72–74^ which may be attributed to the varied dosage and duration of immunosuppressive drugs used in different studies.

For patients with autoimmune diseases, the expert consensus panel recommend the following:

- Eliminate active infectious lesions and avoid exacerbating abnormal immune responses during endodontic therapy.
- Improve oral hygiene and dietary habits to minimize risks of caries or endodontic diseases for patients with autoimmune diseases.

### Neurological/psychiatric disorders

Diagnosing endodontic diseases in patients with neurological/psychiatric disorders can pose a significant challenge. For instance, individuals with autism or Alzheimer’s disease often have social and communication barriers,^75,76^ which may impede their direct communication with dental practitioners. It is advisable for endodontists to engage in discussions with accompanying family members or guardians and place greater reliance on objective results from intraoral and imaging examinations during the diagnostic process.

Careful differential diagnosis should be exercised when dealing with patients exhibiting signs and symptoms of Munchausen syndrome, as they frequently seek medical attention by fabricating illnesses or symptoms, sometimes even intentionally causing harm to themselves or others for sympathy.^77^ The characteristics displayed by such patients include: (1) providing vague self-reported medical history that does not align with clinical examinations or imaging; (2) seeking multiple consultations and treatments for similar or identical dental issues without experiencing improvement or relief; (3) displaying visible signs of self-inflicted injuries within the oral cavity such as ulcers, gum recession, or tooth fractures; (4) exhibiting abnormal or exaggerated reactions toward dental procedures; (5) refusing to cooperate in diagnostic tests and frequently canceling or missing follow-up appointments; (6) demonstrating strong indications of drug-seeking behavior, such as requesting specific medications or higher doses of analgesics.^78^ When Munchausen syndrome is suspected, it is important to approach the situation with sensitivity and avoid directly accusing the patient of dishonesty. This will help preventing the patient from becoming defensive or hostile. Instead, focus on building a trusting relationship with the patient and gaining an understanding of his/her psychological and social challenges. It is crucial to document the patient’s medical history, symptoms, and treatment carefully and objectively while minimizing any unnecessary or invasive procedures.

For patients with epilepsy and planned for endodontic therapy, it is imperative to obtain information from patients or their family members regarding the patient’s seizure history and the psychiatric evaluation results on their mental state and personal behavior management.^79^ A comprehensive assessment should be conducted on the type of seizures, any known triggering factors (such as stress or bright lights), presence of prodromal symptoms before seizures occur, and any history of injury related to seizures.^80^ It is advisable to minimize light stimulation and avoid emotional fluctuations and discomfort during the treatment in order to minimize the risk of a seizure episode.^81^ If a seizure occurs, it is crucial to promptly remove the treatment tray and instruments, clear any foreign objects from the patient’s mouth, recline the dental chair to a supine position, and restrain the patient to prevent fall and injuries if possible.^82^ If a seizure persists for more than 5 min, immediate airway support and administration of oxygen should be provided,^82^ followed by urgent transfer to a comprehensive hospital for emergency treatment. Patients who suffer from seizures may exhibit excessive tooth wear or even tooth fracture,^80^ consequently leading to a less favorable outcome for endodontic therapy.

For patients with Parkinson’s disease, involuntary tremors may impede dental procedures.^83^ It is recommended that dental procedures be scheduled for short durations (not exceeding 45 min) in the morning when their symptoms are milder, and medication is most effective. Additionally, it is advised that patients void their bladder prior to treatment to minimize symptom exacerbation.^84^ Patients with Parkinson’s disease frequently experience tremors that affect their facial muscles and are often prescribed levodopa medication.^85,86^ When using local anesthetics containing epinephrine/epinephrine-like substances, special attention should be paid to Parkinson’s disease patients who are taking levodopa medication as these individuals may exhibit increased sensitivity toward epinephrine-induced changes in blood pressure and heart rate.^87^ After endodontic therapy, it is important to gradually raise the dental chair for patients with Parkinson’s disease to accommodate the autonomic dysfunction associated with the condition and prevent orthostatic hypotension or syncope.^88,89^

For patients with dental phobia, anxiety can be alleviated by providing a comfortable waiting environment, use of relaxing music,^90^ atraumatic restorative treatment (ART),^91^ and observation of non-phobic individuals undergoing dental diagnosis and treatment to reduce fear.^92^ If the patients are unable to cooperate with chairside treatments, and they have passed a general anesthesia assessment, endodontic therapy may be performed under general anesthesia.^93,94^

For patients with neurological/psychiatric disorders, the expert consensus panel recommend the following:

- Comprehensively and prudently collect information and emphasize objective examination results.
- Assess the patient’s mental state before endodontic therapy.
- Avoid inducing or aggravating the patient’s original neurological/psychiatric disorders during treatment procedures, and formulate the emergency plan.
- Pay attention to the influence of medications on endodontic therapy.
- Consider general anesthesia/sedation for patients with severe neurological or psychiatric disorders.

### Cancer-related systemic conditions

Patients with multiple myeloma and breast cancer may have history of using bisphosphonate drugs as adjunct therapy. These patients, particularly those undergone intravenous bisphosphonate therapy, have increased risk of developing bisphosphonate-related osteonecrosis of the jaw (BRONJ) after dental implant surgery or tooth extraction.^95–97^ NSET has been recommended as an alternative to avoid tooth extraction, apical surgery, or dental implant in order to minimize the risk of BRONJ.^98^ Endodontic therapy followed by restoration is a relatively safe for patients with history of using bisphosphonates,^99^ but it should be noted that endodontic therapy itself has also been associated with BRONJ.^100^ Cautions should be exercised during rubber dam placement to prevent soft tissue injury which could increase the risk of BRONJ.^101–103^ Infection resulting from debris extrusion through the apical foramen during root canal treatment may also lead to the development of BRONJ.^104^ To reduce oral bacterial load before initiating treatment, patient may be advised to rinse their mouth with chlorhexidine for 1 min.^105^ Thorough removal of carious tissues prior to root canal treatment may also be beneficial.^98^ The prophylactic use of antibiotics remains controversial. However, for patients who have received intravenous bisphosphonates or have taken oral bisphosphonates exceeding 3 years duration, and those who have poor overall health conditions (such as renal failure, diabetes, or immunodeficiency) and are concurrently using chemotherapy drugs or steroids, and for patients who are planned to receive root canal treatment for multiple teeth, prophylactic antibiotics may be considered.^98^ Bisphosphonates can impact bone metabolism and impede alveolar bone healing. For patients with persistent periapical lesions after root canal treatment, close monitoring should be conducted while also considering the possibility of BRONJ. In case of root canal treatment failure in patients with history of bisphosphonate use, patient evaluation and risk assessment are recommended before deciding retreatment, apical surgery, or tooth extraction.

Radiation therapy for head and neck cancer (HNC) can lead to a reduction in the number of osteocytes, osteoblasts and endothelial cells, and decreased blood flow,^106,107^ thus potentially increasing the risk of pulp necrosis in teeth. However, a recent systematic review concluded that radiation therapy for HNC significantly alters dental pulp responses, but does not cause pulp necrosis.^108^ Radiation therapy for HNC can damage salivary glands, leading to reduced saliva secretion and increased risk of dental caries.^109^ It is recommended that patients undergo endodontic therapy for symptomatic non-vital teeth at least 1 week prior to radiation therapy. Treatment for asymptomatic non-vital teeth can be postponed.^38^

Chemotherapy for cancer increases the susceptibility of patients to infections. Before endodontic therapy in patients undergoing chemotherapy, it is essential to evaluate the patient’s hematological parameters such as white blood cell count, platelet count, coagulation function and etc., and assess the condition of oral mucosa to determine if there is an increased risk of infection or bleeding.^110^ During endodontic therapy, it is crucial to minimize damage to the periodontal tissues and strictly adhere to aseptic principles to reduce the risk of infection.^111^ Furthermore, patients should be reminded to diligently maintain oral hygiene throughout the course of endodontic therapy.^112^

For patients with cancer-related systemic conditions, the expert consensus panel recommend the following:

- Carefully inquire the specific treatment plan and drug history.
- Emphasize aseptic techniques during endodontic therapy to avoid iatrogenic infections.

### Inflammatory bowel disease

Inflammatory bowel disease (IBD) includes Crohn’s disease and ulcerative colitis, which can affect any segment of the gastrointestinal tract. During endodontic therapy, it is advisable for patients with IBD to avoid the use of NSAIDs for dental pain in order to prevent exacerbation of IBD symptoms. If necessary, acetaminophen can be used as an alternative. Prolonged administration of glucocorticoids in IBD patients (prednisone 20 mg/day or more within the past 9–12 months) may carry a risk of adrenal suppression. In such case, the steroid regimen may need to be increased during invasive endodontic therapy, particularly for anxious patients with challenging preoperative or postoperative pain management, or when a complex or demanding intervention is anticipated.^113^

For patients with IBD, the expert consensus panel recommend the following:

- Be aware of the oral manifestations associated with IBD.
- Avoid the use of NSAIDs.
- Adjust the steroid regimen if necessary.

## Conclusions

The overall health condition of patients significantly influences the diagnosis, treatment, and prognosis of endodontic diseases. Endodontists should possess a comprehensive understanding of the intercorrelations between common systemic diseases and endodontic diseases, as well as a thorough awareness of potential risk for patients with specific systemic diseases during endodontic therapy. Based on currently available evidence, this consensus comprehensively summarizes the impact of cardiovascular disease, diabetes, chronic kidney disease, blood-borne infectious diseases, autoimmune diseases, neurological/psychiatric disorders, cancer-related systemic conditions, and inflammatory bowel disease on the treatment planning, specific procedures and clinical outcome of endodontic therapy. When dealing with patients with multiple systemic diseases, it is important to recognize the limitations of individual medical knowledge reserves and clinical diagnostic techniques. Interprofessional care involving a multidisciplinary team, guided by both evidence-based medicine and health economics, can help implement optimal treatment for both systemic and endodontic conditions, ensuring patient safety and improving the long-term functional preservation of affected teeth.
